# Supplementary material for: Ultrasonic Evaluation of Diaphragm in Patients with Systemic Sclerosis
Source: J Pers Med. 2023 Sep 27;13(10):1441. doi: 10.3390/jpm13101441 (PMC10608128; doi:10.3390/jpm13101441)

Figure S1: Correlations of diaphragmatic mobility during deep breathing with evaluated parameters

Figure S1a: Negative correlation between diaphragmatic mobility during deep breathing and Modified Medical Research Council dyspnea scale (mMRC)

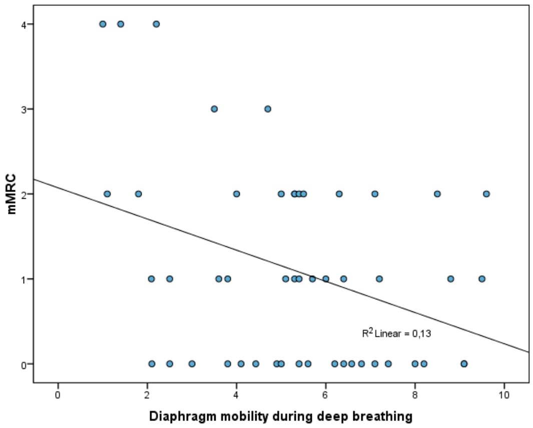

Figure S1b: Negative correlation between diaphragmatic mobility during deep breathing and esophageal diameter on HRCT at location 2

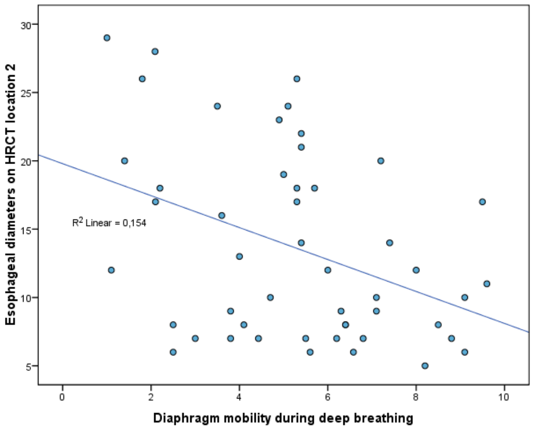

Figure S1c: Negative correlation between diaphragmatic mobility during deep breathing and modified Rodnan skin score (mRSS)

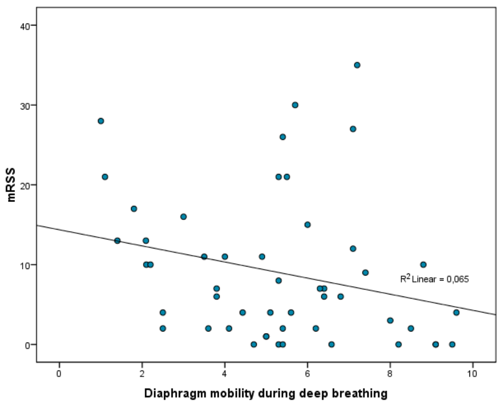

Figure S1d: Positive correlation between diaphragmatic mobility during deep breathing and alveolar volume (VA %)

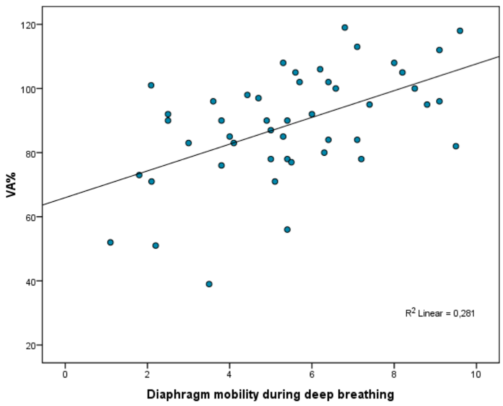

Figure S1e: Positive correlation between diaphragmatic mobility during deep breathing and forced vital capacity (FVC %)

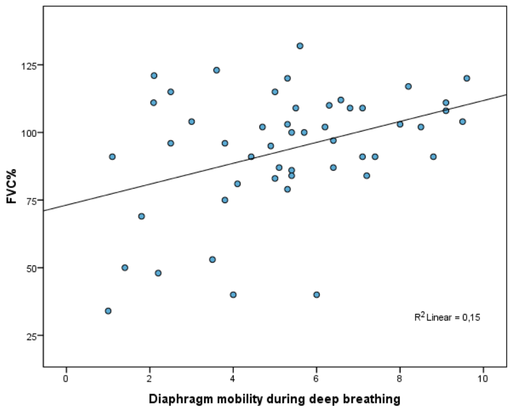

Supplement: Supplementary file 1 [file jpm-13-01441-s001.zip › jpm-2594637-supplementary/Figure S1.pdf]
