# Supplementary material for: Ultrasonic Evaluation of Diaphragm in Patients with Systemic Sclerosis
Source: J Pers Med. 2023 Sep 27;13(10):1441. doi: 10.3390/jpm13101441 (PMC10608128; doi:10.3390/jpm13101441)
Supplement: Supplementary file 1 [file jpm-13-01441-s001.zip › jpm-2594637-supplementary/Table S2.pdf]

Table S2. Warrick score for evaluation of interstitial lung disease

**Severity score**	Grade	**Extent score***	Grade
ground-glass opacities	1	1–3 segments	1
irregularity of the pleura	2	4–9 segments	2
septal/subpleural lines	3	>9 segments	3
honeycombing	4		
subpleural cysts	5		
**Global score** = total severity score + total extent score			

*number of segments involved
