# Supplementary material for: Ultrasonic Evaluation of Diaphragm in Patients with Systemic Sclerosis
Source: J Pers Med. 2023 Sep 27;13(10):1441. doi: 10.3390/jpm13101441 (PMC10608128; doi:10.3390/jpm13101441)
Supplement: Supplementary file 1 [file jpm-13-01441-s001.zip › jpm-2594637-supplementary/Table S3.pdf]

Table S3 Correlations between variables of diaphragmatic ultrasonic evaluation and lung function tests, Warrick score, and esophageal diameters on HRCT

| | Spearman's coefficient of correlation Rho (P-value) | | | | | | | | |
|---|---|---|---|---|---|---|---|---|---|
| | Alveolar volume% | DLCO% | FVC% | Severity Warrick score | Extent Warrick score | Global Warrick score | Esophageal diameter 1 | Esophageal diameter 2 | Esophageal diameter 3 |
| Diaphragm mobility in deep breathing | **0.501 (<0.001)** | 0.273 (0.05) | **0.313 (0.03)** | **–0.414 (<0.001)** | **–0.397 (<0.001)** | **–0.339 (0.02)** | **–0.368 (0.01)** | **–0.366 (0.01)** | –0.182 (0.21) |
| Diaphragm mobility in normal breathing | 0.193 (0.19) | 0.078 (0.59) | –0.099 (0.50) | **–0.309 (0.03)** | –0.192 (0.18) | **–0.342 (0.01)** | –0.177 (0.22) | –0.044 (0.76) | –0.021 (0.88) |
| Diaphragmatic thickening fraction right side | –0.046 (0.76) | **0.313 (0.03)** | –0.001 (0.99) | –0.209 (0.15) | –0.192 (0.18) | –0.157 (0.28) | –0.144 (0.32) | **–0.321 (0.02)** | –0.247 (0.09) |
| Diaphragmatic thickness right side | | | | | | | | | |
| At FRC | 0.154 (0.30) | –0.074 (0.61) | 0.26 (0.07) | 0.228 (0.11) | **0.308 (0.03)** | 0.23 (0.11) | 0.242 (0.09) | **0.34 (0.02)** | 0.276 (0.05) |
| At TLC | 0.09 (0.55) | 0.011 (0.94) | **0.285 (0.04)** | 0.164 (0.25) | 0.219 (0.13) | 0.155 (0.28) | 0.087 (0.55) | 0.214 (0.14) | 0.12 (0.41) |

DLCO-diffusing capacity for carbon monoxide, FVC-forced vital capacity, FEV1-forced expiratory volume in the first second, FRC-functional residual capacity, TLC-total lung capacity
