# Supplementary material for: Ultrasonic Evaluation of Diaphragm in Patients with Systemic Sclerosis
Source: J Pers Med. 2023 Sep 27;13(10):1441. doi: 10.3390/jpm13101441 (PMC10608128; doi:10.3390/jpm13101441)
Supplement: Supplementary file 1 [file jpm-13-01441-s001.zip › jpm-2594637-supplementary/Table S4.pdf]

Table S4. Multivariate regression analysis according to interstitial lung disease

Multivariate logistic regression	ß	P value	OR	95% CI
Esophageal diameter at location 1 (mm)	0.56	0.005	1.76	1.19 do 2.60
Constant	–4.4	0.001	0.01	

ß – regression coefficient
